# Risk factor of postoperative incision infection after plate internal fixation of calcaneal fractures: a retrospective study

**DOI:** 10.1186/s12891-022-06072-4

**Published:** 2022-12-14

**Authors:** Lei Shen, Qiang Wang, Jun Chen, Zhenhuan Jiang

**Affiliations:** Department of orthopaedics, the Yixing People’s Hospital, 75 Road Tongzhenguan, Yixing, Jiangsu China

**Keywords:** Calcaneal fractures, Infection, Risk factor, Plate internal fixation

## Abstract

**Background:**

This study aimed to investigate the risk factors for incision infection after plate internal fixation for calcaneal fractures by a traditional lateral L-shaped approach.

**Methods:**

The clinical data of 302 patients with calcaneal fractures who underwent surgical treatment in our hospital from January 2012 to June 2018 were retrospectively analysed, consisting of 177 males and 125 females. The enrolled patients were aged 21 to 75 years, with a mean age of 47.72 years. According to the Sanders classification, 108 patients were type II, 138 patients were type III, and 56 cases were type IV. A univariate analysis was conducted with sex, age, smoking history, history of diabetes, cause of injury, Sanders type, tension blisters, time from injury to surgery, preoperative haemoglobin, preoperative albumin, operation time, and bone grafting as possible risk factors. The factors with statistically significant differences were selected for multivariate binary logistic regression analysis. The clinical cut-off values of these risk factors were calculated using characteristic curves.

**Results:**

The follow-up lasted for at least 1 year for all patients, with a mean follow-up time of 15.8 months. The results demonstrated 7.9% (24/302) infection rate after plate internal fixation of calcaneal fractures by the traditional lateral L-shaped approach. Univariate analysis showed that a history of diabetes, preoperative albumin, preoperative haemoglobin, time from injury to surgery, and operation time were correlated with incision infection (*p* < 0.05). Additionally, multivariate regression analysis indicated that a shorter time from injury to surgery (*OR* = 1.475, *95% CI*: 1.024-2.125, *p* = 0.037), lower preoperative albumin (*OR* = 1.559, *95% CI*: 1.191-2.041, *p* = 0.001), and longer operation time (*OR* = 1.511, *95% CI*: 1.219-1.874, *p* < 0.001) were risk factors for postoperative incision infection, and their cut-off values were 10.5 days, 38.5 g/L, and 84.5 minutes, respectively.

**Conclusion:**

Longer preoperative stay and operation time were two risk factors for postoperative incision infection. However, lower preoperative albumin level is the highest risk factor in this study.

**Trial registration:**

The trial was registered in the China Clinical Trial Registry (ChiCTR2100047038).

Calcaneal fractures account for approximately 1-4% of all fractures in adults, and 60-80% are intra-articular [1]. Despite no consensus on the treatment of displaced intra-articular calcaneal fractures, in the absence of surgical contraindications, anatomical reduction and fixation should be performed if the calcaneal articular surface collapses more than 2 mm to avoid limited ankle motion and traumatic arthritis of the subtalar joint [2, 3]. Incision infection comprises one of the most frequent complications following a calcaneal fracture operation, involving deep structures such as bone, deep fascia, and implants, which may eventually lead to the formation of infection biofilms, osteomyelitis, scar formation, and even the need to remove the internal fixation. Therefore, it is particularly necessary to prevent incision infection after the calcaneal fracture operation. This study retrospectively analysed the clinical data of patients with calcaneal fractures treated by plate internal fixation via a traditional lateral L-shaped approach at our hospital from January 2012 to June 2018 to identify the risk factors for postoperative incision infection, thus providing suggestions for clinical treatments.

## Methods

### Infection classification and diagnostic criteria

In this study, infection was classified into superficial infection and deep infection according to the US Centers for Disease Control and Prevention criteria [4].

### Patient inclusion and exclusion criteria

A retrospective analysis was performed for patients with calcaneal fractures who received plate internal fixation surgery via a traditional lateral L-shaped approach at our hospital from January 2012 to June 2018. Inclusion criteria: 1) age ≥ 18 years; 2) patients with Sanders II, III, and IV calcaneal fractures. Exclusion criteria: 1) patients with Sanders I calcaneal fractures; 2) patients who received fixation via other approaches; 3) patients who received other internal fixation; 4) patients with other complicating fractures; 5) patients with severe neurovascular injuries in the operated side lower extremity; 6) patients with local or systemic infection before surgery; 7) patients with liver, renal or autoimmune diseases.

### Perioperative management

#### Management from admission to surgery

After admission, all patients were requested to elevate the injured limb above the heart level; at the same time, the swelling was alleviated by an ice compress on the heel of the injured limb and intravenous administration of 20% mannitol until the day before surgery. Patients received prophylactic treatment to prevent deep vein thrombosis of the lower extremities based on the Caprini score. For tension blisters caused by swelling, the blister wall was maximally maintained to be intact. The fluid of large blisters was aspirated after full disinfection, and then sterile dressing and elastic bandage were used. All patients underwent a comprehensive evaluation of fractures by preoperative lateral and axial X-ray examinations of the calcaneus, CT scan, and three-dimensional reconstruction. Surgery was conducted when wrinkles occurred on the heel skin following sufficient detumescence [5].

#### Surgical procedures

All operations were performed by orthopaedic surgeons with more than 15 years of experience. Cephalosporin antibiotics were routinely used preventively 30 minutes before surgery, while clindamycin was adopted for infection prevention in those allergic to β-lactam. Principles of surgical treatment: 1) aseptic operative procedures; 2) soft tissue protection; 3) anatomical reduction of the calcaneal articular surface; 4) restoration of geometric parameters of the calcaneus, such as length, width, and height; and 5) correction of varus and valgus of the calcaneus. All patients were placed in the lateral decubitus position, and lower-extremity balloon tourniquets were used during the operation.

The surgical incision starts from the lateral edge of the Achilles tendon at the posterior tip of the lateral malleolus, extends along the posterior edge of the fibula to the junction of plantar and dorsalis, horizontally forwards to the junction of plantar and lateral plantar skin, and then turns to the base of the fifth metatarsal bone. The skin and subcutaneous tissues were dissected until the bone surface, clinging to which the skin flap was peeled off to expose the lateral wall of the calcaneus. Multiple Kirschner wires were used for the reduction and fixation of the fracture fragments to restore the flatness of the calcaneal articular surface. Allogenic bone grafting was performed for patients with obvious bone defects. After satisfactory fracture reduction through C-arm fluoroscopy, an appropriately sized calcaneal plte (Trauson Medical Instrument Co., Ltd., Changzhou, Jiangsu, China, or Shandong WEGO Group Co., Ltd., Weihai, Shandong, China) was selected and fixed with an appropriate number of screws. Fracture reduction was reassessed by fluoroscopy. After the surgical area was fully rinsed, a drainage tube was placed in the incision. The incision was sutured layer by layer without tension. A sterile dressing covered with an elastic bandage was utilized for pressure dressing.

#### Postoperative management

With the injured limbs elevated above the heart level, all patients were given an intravenous administration of 20% mannitol to reduce swelling until 3-7 days after surgery. The wounds were cleaned, and the dressings were changed to keep the incision dry. Additionally, patients underwent red light physiotherapy on the incision twice a day, and the drainage tube was extracted depending on the wound exudation. All patients were treated with prophylactic antibiotics within 24 hours following surgery. Based on the Caprini score, prophylactic treatments for deep vein thrombosis of the lower extremities were performed. Subtalar joint functional exercise can be started if the incision heals well, and weight-bearing exercise can be started after X-ray examination 3 months following surgery. Patients with deep infection were required to exercise non-weight-bearing rehabilitation for at least 3 months, only including ankle flexion and extension. The timing of weight-bearing exercise should be determined according to the imaging review.

#### Outcome measures

The main outcome measures were as follows: 1) basic clinical characteristics: age, sex, causes of injuries, fracture classification, smoking history, history of diabetes, preoperative haemoglobin, preoperative albumin, and tension blisters; 2) surgical data: time from injury to surgery, operation time, and bone grafting; and 3) evaluation of postoperative incision infection signs: incision swelling, exudation, sinus formation, inflammatory indicators, and bacteriological culture of incision secretions.

### Statistical analysis

IBM SPSS 24.0 statistical software was utilized for statistical analysis. Data normality was examined by the Shapiro–Wilk test. Normally distributed continuous data were analyzed for statistical differences using the independent samples t-test, non-normally distributed data were analyzed by the Mann-Whitney test. Enumeration data were analysed by χ2 test or Fisher’s exact test. A multivariable analysis by binary logistic regression was performed for risk factors with significant differences after an univariable analysis. *p* < 0.05 was deemed to be statistically significant.

## Results

### Univariable analysis of risk factors for infection

All the enrolled patients were followed up for at least 1 year, with a mean follow-up time of 15.8 months. The patients were divided into an infection group (*n* = 24; 7.9%) and a noninfection group (*n* = 278; 92.1%). There were 10 cases of deep infection in the infection group, and their incisions were well healed after thorough debridement and flap coverage without serious complications such as fracture nonunion or talocalcaneal joint fusion. After daily cleaning, dressing changes, red light physiotherapy, and prolonged antibiotic use, 14 patients with superficial infections did not develop deep infections, and their incisions were well healed. The univariate analysis results showed significant differences concerning the incidence of preoperative tension blisters, smoking history, history of diabetes, preoperative haemoglobin, preoperative albumin, time from injury to surgery, and operation time between the two groups (*p* < 0.05). No significant differences were observed regarding age, sex, cause of injury, fracture classification, anaesthesia method, or intraoperative bone grafting between the two groups (*p* > 0.05) (Table 1).

**Table 1:** The Univariate analysis of all factors

	Infection Group(***N*** = 24)	Non-infection Group(***N*** = 278)	Statistics	***p*** value
**Age(years)**	43.125 ± 16.028	48.115 ± 15.126	−1.543	0.124
**Sex**				
Male	14	163	0.001	0.977
Female	10	115		
**Injury reason**				
Falling	13	163	3.289	0.193
Accident	3	62		
Squeezing	8	53		
**Sanders type**				
II	6	102	2.431	0.297
III	11	127		
IV	7	49		
**Blister**				
Yes	13	145	0.036	0.850
No	11	133		
**Smoking**				
Yes	10	123	0.06	0.807
No	14	155		
**Diabetes**				
Yes	12	80	4.698	0.03
No	12	198		
**Preoperative hemoglobin**	33.792 ± 3.878	41.374 ± 4.986	−7.258	<0.001
**Preoperative albumin**	110.750 ± 6.173	115.252 ± 8.930	−2.418	0.016
**Anesthesia**				
Generl anesthesia	11	102	0.789	0.375
Lumbar anesthesia	13	176		
**Time from injury to operation**	11.042 ± 3.085	13.407 ± 2.242	−4.795	<0.001
**Operation Time(minutes)**	95.410 ± 8.871	77.917 ± 5.941	−9.471	<0.001
**Bone graft**				
Yes	17	182	1.863	0.172
No	7	96		

### Multivariable analysis of risk factors for infection by binary logistic regression

**Fig. 1:**
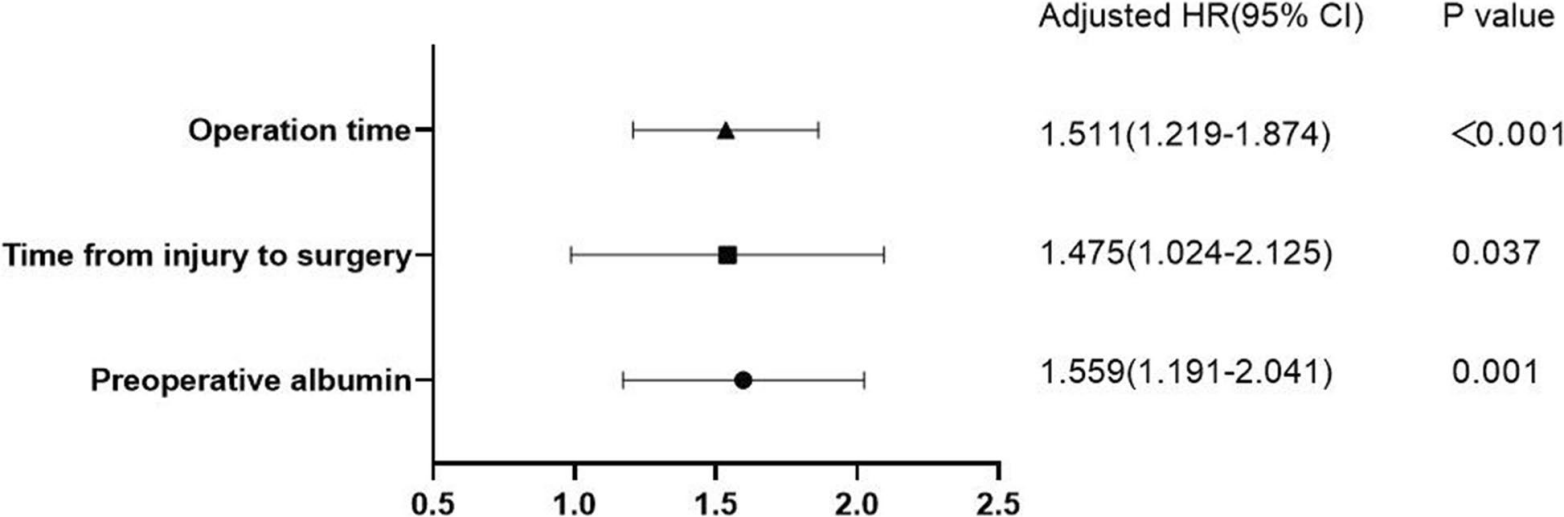
Forest plots of regression analysis results

A history of diabetes, preoperative haemoglobin, preoperative albumin, the time from injury to surgery, and the operation time were listed as independent variables, and infection was regarded as the dependent variable. The results of binary logistic regression analysis showed that a shorter time from injury to surgery, longer operation time, and lower preoperative albumin were risk factors for postoperative incision infection (Table 2). Forest plots of regression analysis results were plotted to indicate the effect size of each factor (Fig. 1).

**Table 2:** The binary logistic regression analysis of the risk factors

Factors	***B***	***SE***	***Wald***	***p***	***OR***	95% ***CI***	
						***Lower***	***Upper***
**Time from injury to surgery**	0.389	0.186	4.35	0.037	1.475	1.024	2.125
**Operation time**	0.413	0.11	14.144	<0.001	1.511	1.219	1.874
**Preoperative albumin**	0.444	0.137	10.457	0.001	1.559	1.191	2.041
**Diabetes**	1.188	0.882	1.816	0.178	3.28	0.583	18.466
**Preoperative hemoglobin**	0.087	0.068	1.673	0.196	1.091	0.956	1.246

### Clinical threshold values of risk factors

**Fig. 2:**
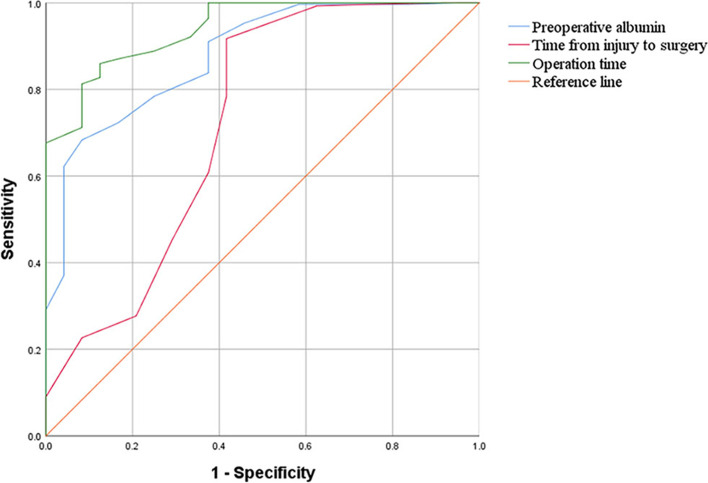
The ROC curves of risk factors

Receiver operating characteristic (ROC) curves were plotted to estimate the clinical threshold value of each risk factor. The results demonstrated that the clinical threshold value of the time from injury to surgery was 10.5 days with a sensitivity of 91.7% and specificity of 58.3%, the clinical threshold value of operation time was 84.5 minutes with a sensitivity of 86.0% and specificity of 87.5%. The preoperative albumin was 38.5 g/L with 68.3% sensitivity and 91.7% specificity 91.7% (Fig. 2).

## Discussion

### Current situation of postoperative incision infection for calcaneal fractures

Existing studies have shown that surgical treatment of displaced calcaneal fractures can achieve favourable therapeutic effects [6]. Still, it correlates with an incidence of postoperative incision infection up to 15.4% [7–9], which is one of the most common complications after internal fixation of calcaneal fractures. Although there have been various surgical approaches for internal fixation of calcaneal fractures in the past 30 years, the lateral L-shaped extension approach remains the most frequently applied method for internal fixation [10]. Through this surgical approach, the soft tissue capsule is opened to expose the fracture site as well as the collapsed and compressed articular surface. Upon poor postoperative incision healing, cracking and exposure of deep structures, including bone, deep fascia, and implants, may develop until infection, resulting in devastating outcomes. The indication for patients with deep infection of whether to remove the internal fixation device is based on the invaded severity of the infection. If a sinus tract is discovered deep to the bone surface, the internal fixation should be removed to avoid residual biofilm infection on the plate surface. Otherwise, the infection can be controlled by adequate debridement and effective antibiotic treatment. Current studies have illustrated nonsurgical factors related to incision infection after internal fixation of calcaneal fractures (higher body mass index, history of diabetes, smoking history, etc.) and surgical-related factors (intraoperative lateral peroneal artery injury, earlier surgical intervention, inexperienced operators, etc.) [11–14]. Yet, most of them involve univariate analysis. Therefore, we conducted a retrospective study incorporating multiple factors by logistic regression analysis. Our study offered data demonstrating an infection rate of 7.9% following internal fixation of calcaneal fractures. The time from injury to operation < 10.5 days, preoperative albumin value < 38.5 g/L, and operation time > 84.5 minutes were determined to be risk factors for postoperative incision infection (*p* < 0.05).

### Analysis of risk factors and corresponding therapeutic strategies

Axial violence represents the most common damage mechanism of calcaneal fractures. However, due to the special anatomical structure around the calcaneus, high-energy trauma and relatively thin surrounding soft-tissue coverage can lead to severe soft-tissue damage and swelling, so preoperative soft-tissue management and appropriate timing of surgery are particularly important. Early surgical treatment, especially surgical intervention within 3 days following injury, may lead to aggravation of swelling, poor postoperative incision healing and even infection, and other complications when soft tissue swelling is at its peak. For more swollen patients, heel ice compress and elevation of the affected limbs are applied to alleviate the swelling, and necessary drugs such as 20% mannitol are given to reduce swelling. It should be noted that 1-10% of patients with calcaneal fractures may develop compartment syndrome [15]. If the patient shows persistent and aggravated heel pain, soft-tissue swelling, and loss of skin sensation, early incision decompression is required to reduce sequelae. If tension blisters occur around the incision, we try to retain the blister wall since the blister fluids contain opsonin and immune cells, exerting anti-infection effects; at the same time, the cyst wall is a natural wound dressing that can protect the blister bed from the outside. The fluid in the large blisters should be aspirated after thorough disinfection, and then a sterile elastic bandage should be utilized for pressure dressing. The univariate analysis in the study showed no significant difference in the incidence of tension blisters between the two groups (54.17% vs. 52.16%, *p* = 0.850), which was closely related to our preoperative aseptic management for blisters. Regarding the timing of surgery, it is currently advocated that surgery performed after the skin folds on the heel causes low rates of complications [16]. However, Ho et al. thought that an experienced surgeon can beperform early surgery for patients who have no potential risk factors for incision infection, which will not increase the incidence of incisional complications [13]. The appropriate time for surgical intervention is the presence of skin folds rather than the complete elimination of soft tissue oedema because it may take a long time to fully eliminate soft tissue oedema, and haematoma mechanization around fracture may affect the operating procedures during the waiting time for detumescence. In this study, the duration from injury to surgery was significantly shorter in the infection group than in the noninfection group (OR = 1.475; 95% CI: 1.024-2.125; *p* = 0.037). The ROC curves showed 10.5 days as the clinical threshold of the duration from injury to surgery, with a sensitivity of 91.7% and specificity of 58.3%.

The length of operation is closely related to adequate preoperative preparation, operative experience, and intraoperative cooperation. The surgical treatment of calcaneal fractures is also an invasive method that induces certain damage to the soft tissues. The lateral L-shaped extended incision that requires opening the lateral calcaneal flap and full exposure of the fracture site is utilized for patients with Sanders type III or IV fractures to obtain a more intuitive surgical perspective. However, a longer operation time and a longer time of pulling the skin flap will indirectly affect postoperative incision healing, even leading to incision infection. The study conducted by Koski et al. noted that the long duration of surgery is a dangerous factor for postoperative incision infection [17]. Similarly, the work of Al-Mudhaffar et al. showed that the operation time of patients with postoperative incision infection after calcaneal fractures was longer than that of noninfected patients, showing a mean increase of 39 minutes [18]. Zhou et al. suggested that tourniquet use during surgery can shorten the operation time by 4.8 minutes [19]. Consistently, Zhang et al. demonstrated that using a tourniquet leads to a mean reduction of 4.6 min in operation time [20]. Therefore, a lower extremity balloon tourniquet was utilized during complex calcaneal fracture surgery in our hospital to ensure a clear surgical field of vision, reduce intraoperative bleeding, and shorten the operation time. Compared to the noninfection group, the operation time increased by 17.49 minutes in the infection group (OR = 1.511; 95% CI: 1.219-1.874; *p* < 0.001). Meanwhile, our ROC curves revealed that the clinical cut-off value of the operation time was 84.5 minutes, and its sensitivity and specificity were 86.0 and 87.5%, respectively.

The negative nitrogen balance induced by trauma and surgery is proportional to the degree of injury [21], so normal nutritional status is essential to maintain the healing ability of skin and soft tissues and to prevent infection [22]. The albumin level represents the clinical haematological indicator most directly linked to the nutritional status of patients. Albumin is regarded as one of the major components of human plasma, and malnutrition is usually defined as an albumin level under 3.5 mg/dl [23]. Bohl et al. found that malnutrition evoked by hypoalbuminemia could affect collagen synthesis and reduce fibroblast proliferation, resulting in poor wound healing [24]. Yi et al. considered that hypoalbuminemia would attenuate the inflammatory response against infection, thus affecting incision healing [25]. In this study, the mean albumin value showed a marked reduction of 7.582 g/L in the infection group compared to the noninfection group (OR = 1.559, 95% CI: 1.191-2.041, *p* = 0.001), and the results of the ROC curves revealed a clinical cut-off value of 38.5 g/L, and its sensitivity and specificity were 68.3 and 91.7%, respectively.

Incision healing is highly correlated with a constant and sufficient oxygen supply. Haemoglobin constitutes the main carrier of oxygen and carbon dioxide in the human body. Oxygen can offer bioenergy, such as adenosine triphosphate, catalyse the production of reactive oxygen species, and mediate collagen components, exerting a crucial role in the incision healing process [26]. High haemoglobin levels can ameliorate hypoxia, reduce hypoxia-induced apoptosis, and promote wound healing, effectively preventing wound necrosis and infection. The univariable analysis indicated a lower haemoglobin level in the infection group (*p* < 0.001). Yet, the regression analysis exhibited no statistical correlation between the low haemoglobin level and incision infection (*p* = 0.196).

Recent studies have suggested that diabetic patients may develop pathological stenosis and occlusion in the blood vessels, resulting in poor blood supply in distal limbs, increased blood viscosity, and impaired red blood cell oxygen transport. Additionally, diabetes limits the function of human immune cells, leading to impaired innate and adaptive immune responses, which increases the risk of various infections [27, 28]. Endara et al. revealed a significant association between blood glucose and postoperative incision infection, and a positive correlation between good perioperative glycaemic control and wound healing in diabetic patients who underwent surgery [29]. If the incision does not heal well after calcaneal fractures, there is a great risk of incision infection, inducing devastating outcomes. Therefore, this imposes an extreme challenge on the treatment of diabetic patients with calcaneal fractures. Rammelt et al. proposed severe insulin-dependent diabetes as a contraindication for open reduction and internal fixation [30]. The univariate analysis of this study demonstrated that a history of diabetes was a risk factor for postoperative incision infection (*p* = 0.03). However, the regression analysis offered data showing no statistical relationship between a history of diabetes and incision infection (*p* = 0.178), which may be associated with good perioperative glycaemic control. It is of great necessity to control the preoperative fasting blood glucose level below 10.0 mmol/L. If the effect of oral drugs is limited, insulin injection can be adopted for blood glucose control.

The results of this study did not suggest intraoperative implantation of allografts to be a risk factor for incision infection after internal fixation of calcaneal fractures. Intraoperative bone grafting is mainly used to provide mechanical support and induce osteogenesis, but whether intraoperative bone grafting is necessary remains controversial. According to the study of Singh et al., although patients with bone grafts show advantages in terms of Böhler angle and time of full weight-bearing, no significant difference is observed in functional outcomes and complications [31]. A meta-analysis conducted by Pan et al. suggested that intraoperative bone grafting increased the risk of postoperative wound infection and other complications (OR = 1.74, *p* < 0.01) [32]. Our univariate analysis results showed that allogeneic bone grafting during surgery did not increase the risk of postoperative wound infection (*p* = 0.172), which potentially correlated with more stable fracture fixation, reduced fracture micromotion, reduced exudation at the fracture site, and a low risk of subcutaneous haematoma after bone grafting. Subcutaneous haematoma is one of the principal causes of postoperative incision infection. Because bone grafting does not increase the incidence of infection in open reduction and internal fixation of calcaneal fractures, we consider that the use of bone grafting during surgery should be determined depending on the actual bone defect.

### Limitations and prospective

This study also has some limitations. First, a retrospective study offers a relatively low level of evidence, there may have some inevitable management differences among these cases. In addition, multiple factors are involved in incision infection following calcaneal fracture surgery. However, this study did not discuss other potential risk factors, such as different surgical approaches, incision suture methods, intraoperative bleeding, implant species, etc. Furthermore, differences may exist in the degree of trauma and treatment process for the calcaneal fractures of different Sanders types. Thus, a large-scale, randomized, controlled study io different types of calcaneal fractures is still necessary for clarification of the relevant risk factors.

## Conclusion

In our study, a postoperative incision infection rate of 7.9%(24/302) was found after treating calcaneal fractures using plate internal fixation via the traditional lateral L-shaped approach. Additionally, this study linked the time from injury to operation < 10.5 days, preoperative albumin value < 38.5 g/L, and operation time > 84.5 minutes to postoperative incision infection. We suggested that these factors should be considered carefully by clinicians in the diagnosis and treatment process. At the same time,we shuld also take the usage of mannitol, the incision length and the internal fixation type into consideration.

## Data Availability

The datasets generated and analysed during the current study are not publicly available due to limitations of ethical approval involving the patient data and anonymity but are available from the corresponding author on reasonable request.
